# Annotation of cis-regulatory-associated histone modifications in the genomes of two Thoroughbred stallions

**DOI:** 10.3389/fgene.2025.1534461

**Published:** 2025-02-27

**Authors:** Alexa M. Barber, Nicole B. Kingsley, Sichong Peng, Elena Giulotto, Rebecca R. Bellone, Carrie J. Finno, Ted Kalbfleisch, Jessica L. Petersen

**Affiliations:** ^1^ University of Nebraska Medical Center, Eppley Institute for Research in Cancer and Allied Diseases, Omaha, NE, United States; ^2^ Department of Animal Science, University of Nebraska-Lincoln, Lincoln, NE, United States; ^3^ Department of Population Health and Reproduction, School of Veterinary Medicine, University of California-Davis, Davis, CA, United States; ^4^ Veterinary Genetics Laboratory, Department of Population Health and Reproduction, School of Veterinary Medicine, University of California-Davis, Davis, CA, United States; ^5^ Department of Biology and Biotechnology, University of Pavia, Pavia, Italy; ^6^ Department of Veterinary Science, University of Kentucky, Lexington, KY, United States

**Keywords:** horse, ChIP-seq, FAANG, functional annotation, histone modifications

## Abstract

The Functional Annotation of Animal Genomes (FAANG) consortium aims to annotate animal genomes across species, and work in the horse has substantially contributed to that goal. As part of this initiative, chromatin immunoprecipitation with sequencing (ChIP-seq) was performed to identify histone modifications corresponding to enhancers (H3K4me1), promoters (H3K4me3), activators (H3K27ac), and repressors (H3K27me3) in eight tissues from two Thoroughbred stallions: adipose, parietal cortex, heart, lamina, liver, lung, skeletal muscle, and testis. The average genome coverage of peaks identified by MACS2 for H3K4me1, H3K4me3, and H3K27ac was 6.2%, 2.2%, and 4.1%, respectively. Peaks were called for H3K27me3, a broad mark, using both MACS2 and SICERpy, with MACS2 identifying a greater average number of peaks (158K; 10.4% genome coverage) than SICERpy (32K; 24.3% genome coverage). Tissue-unique peaks were identified with BEDTools, and 1%–47% of peaks were unique to a tissue for a given histone modification. However, correlations among usable reads, total peak number, and unique peak number ranged from 0.01 to 0.92, indicating additional data collection is necessary to parse technical from true biological differences. These publicly available data expand a growing resource available for identifying regulatory regions within the equine genome, and they serve as a reference for genome regulation across healthy tissues of the adult Thoroughbred stallion.

## 1 Introduction

One of the fundamental goals of genetic research in agricultural species is to associate genomic variation with phenotypic traits of interest. The generation of reference genomes for many species was critical in facilitating genome-wide association studies; however, genomic annotation primarily based on transcriptomic data limits the success of these studies. With protein coding sequences comprising less than 3% of the genome, it is unsurprising that nearly 90% of human trait-associated variants fall outside protein coding sequence (Hindorff et al., 2009; The ENCODE Project Consortium, 2012; Maurano et al., 2012). To address these shortcomings in the annotation of the human reference genome, the Encyclopedia of DNA Elements (ENCODE) project was established with the goal of identifying all functional elements in the human genome.

In the past two decades, the ENCODE project has attributed function to over 80% of the human genome, indicating a large presence of functional elements outside of coding DNA. Cis-regulatory elements aid in maintaining transcriptional programming and include promoters, enhancers, and silencers. In contrast to the roughly 20,000 annotated protein coding genes, the ENCODE project identified nearly 400,000 enhancer and 70,000 promoter regions in the human genome (The ENCODE Project Consortium, 2012). The functional annotation resulting from the ENCODE project and other epigenomic studies has empowered subsequent research that has elucidated the impact of non-coding variants on diseases. Single nucleotide variants (SNVs) and differential methylation within enhancers and promoters have been associated with disorders, such as Alzheimer’s disease, multiple sclerosis, diabetes, congenital heart disease, and other complex diseases (reviewed by van der Lee et al., 2020; Claringbould and Zaugg, 2021). Furthermore, structural variants in cis-regulatory regions can result in enhancer hijacking and disruption of topologically associated domains (TADs) which are frequently implicated in cancer development (Adkemir et al., 2020; Zhang et al., 2022). These studies demonstrate the importance of examining non-coding regions of the genome and annotation of cis-regulatory regions.

With inspiration from the landmark discoveries of the ENCODE project, the Functional Annotation of Animal Genomes (FAANG) initiative was established to functionally annotate the genomes of domesticated animal species and improve the understanding of the genotype-to-phenotype link (The FAANG Consortium, 2015; Tuggle et al., 2016). Part of this effort includes annotating cis-regulatory associated elements using chromatin immunoprecipitation and sequencing (ChIP-seq). ChIP-seq captures the location of histone protein modifications involved in gene regulation, such as methylation (me) and acetylation (ac) of lysine residues on the H3 protein (H3K). The FAANG initiative prioritized ChIP-seq of four histone modifications associated with enhancers (H3K4me1), promotors (H3K4me3), active genomic regions (H3K27ac), and repressed genomic regions (H3K27me3) as core assays (Giuffra et al., 2019). The equine FAANG project previously characterized these four histone modifications in 11 tissues from two Thoroughbred mares (Kingsley et al., 2020; Kingsley et al., 2021). Hundreds of thousands of cis-regulatory associated elements were identified, with 4%–32% of peaks in a given tissue being unique to that tissue (Kingsley et al., 2020). With many genes being differentially expressed between sexes (Lopes-Ramos et al., 2020), the activity of regulatory elements is likely to also differ between sexes. Indeed, differences in histone modifications have been observed between sexes in other species demonstrating the need for annotation of regulatory elements in both sexes (Shen et al., 2015; Keiser and Wood, 2019; Kfoury et al., 2021). Given the detailed analysis performed in mares and no such efforts to focus solely on stallions, this project aimed to characterize histone modifications in the tissues of two adult Thoroughbred stallions to complement the extensive analysis and annotation of cis-regulatory elements in Thoroughbred mares previously published by Kingsley et al. (2020), Kingsley et al. (2021). The incorporation of these data into analyses within and between sexes was included in Peng et al. (2023); the methods of analysis of the cis-regulatory elements in the stallions, and the characterization of ubiquitous and tissue-specific peaks, however, was not previously described. These data continue to contribute to studies of sex-specific and cross-species evaluation of genome function.

## 2 Materials and methods

### 2.1 Chromatin extraction and immunoprecipitation

Tissues from two Thoroughbred stallions (ECA_UCD_AH3 [AH3] and ECA_UCD_AH4 [AH4]), were obtained from the equine FAANG Biobank. Complete veterinary reports are available for both stallions in Donnelly et al. (2021). Stallions AH3 and AH4 were aged three and four, respectively, at the time of donation. Stallion AH3 suffered a career-ending musculoskeletal injury in race training prior to donation. Stallion AH4 was not race trained and was the son of the reference genome donor, Twilight. Tissue samples that were prioritized for chromatin immunoprecipitation and sequencing (ChIP-seq) included abdominal adipose, parietal cortex (brain), left ventricle (heart), lamina, liver, lung, longissimus dorsi (muscle), and testis. Collected tissues were flash frozen in liquid nitrogen and stored at −80°C (Donnelly et al., 2021). ChIP preparation and sequencing was performed by Diagenode using their ChIP-seq Profiling Service (Diagenode, Cat# G02010000, Liège, Belgium). Chromatin was extracted and prepared using the iDeal ChIP-seq kit for Histones (Diagenode Cat# C01010059). Tissue samples were first homogenized using a Tissue Lyser II (Qiagen, Germany) and fixed in 1% formaldehyde to crosslink histone proteins with DNA. Chromatin was sheared using a Bioruptor Pico (Diagenode, Cat# B01060001, Liège, Belgium) in 30 s burst to achieve a targeted fragment size of 200 bp. A temperature of 4°C (10°C for adipose) was maintained during shearing (Bioruptor water cooler). The optimization of these parameters had been previously completed at Diagenode for equine adipose, parietal cortex, left ventricle (heart), lamina, liver, lung, and skeletal muscle as part of the equine FAANG project published by Kingsley et al. (2020). Optimization of chromatin extraction, ChIP, and library preparation for testis was performed for this study. Information regarding the homogenization, fixation, and shearing of each sample is reported in Supplementary Table 1. After crosslink reversal and DNA purification, shearing was assessed using the High Sensitivity NGS Fragment Analysis Kit (DNF-474) on an Agilent Fragment Analyzer (Santa Clara, CA, United States).

Immunoprecipitation (IP) of H3K27ac, H3K27me3, H3K4me1, and H3K4me3 histone marks was performed using the IP-Star Compact Automated System (Diagenode, Cat# B03000002, Liège, Belgium) in all samples except muscle, which was done manually due to low chromatin retrieval. IP of IgG served as a negative control across samples, and 1% of chromatin from each sample was set aside prior to IP for an input sample that serves to correct for background noise in downstream analysis. The amount of antibody used to precipitate each histone mark and IgG differed across tissues and was previously optimized (Kingsley et al., 2020) (Supplementary Table 2).

### 2.2 Library preparation and sequencing

Libraries for the input and ChIP samples for each of the four histone marks were prepared using the MicroPlex Library Preparation Kit v3 (Diagenode Cat# C05010001). Seven to thirteen PCR cycles were used to amplify libraries and achieve appropriate concentrations for sequencing. Libraries were double size-selected for fragments with insert sizes of ∼200 bp using Agencourt^®^ AMPure^®^ XP (Beckman Coulter, Brea, CA, United States) and quantified with the Qubit dsDNA HS Assay Kit (Thermo Fisher Scientific, Q32854, Waltham, MA, United States). Libraries were sequenced as 50bp, paired-end reads on an Illumina HiSeq 4,000 platform (San Diego, CA, United States) to a target depth of 100 million raw reads for H3K27me3 (broad mark) and 50 million raw reads for H3K327ac, H3K4me1, and H3K4me3 (narrow marks) and input samples as determined by the data presented by Kingsley et al. (2020), Kingsley et al. (2021).

### 2.3 Library mapping and read filtering

Adapters were removed and reads trimmed using Trim-Galore/0.6.5 (Krueger, 2019). Reads were mapped to EquCab3.0 with BWA-mem/0.7.17 (Li, 2013). Samtools/1.9 (Li et al., 2009) was employed to mark PCR duplicates and remove read pairs that were unmapped, non-primary alignments, optical duplicates, or had a mapping alignment quality score (MAPQ) of less than 30 prior to peak calling. The targeted usable fragment counts were 45 million for H3K27me3 and 20 million for the remaining marks and input samples as outlined in the ENCODE project (https://www.encodeproject.org/chip-seq/histone/). The H3K27ac adipose sample from ECA_UCD_AH3 had less than half of the targeted usable fragments, so an additional library was prepared and sequenced. The filtered reads from both rounds of sequencing were merged for downstream analysis.

### 2.4 Peak calling and signal tracks

Peaks, representing regions of read pileup, were identified using the pipeline established by Kingsley et al. (https://faang.org/ebi/ftp.ebi.ac.uk/faang/ftp/protocols/analyses/UCD_SOP_processing_and_analyzing_equine_PE_ChIP_data_20201230.pdf). MACS2/2.1.1 (Zhang et al., 2008) was used to call peaks across all four histone marks with a false discovery rate (FDR) cutoff of 0.01 for H3K4me3 and H3K27ac and an FDR cutoff of 0.05 for H3K4me1 and H3K27me3. The “--broad” flag and a broad cutoff of 0.1 were employed for calling H3K27me3 peaks in MACS2. Fold-enrichment (FE) over the input control was determined for each sample in MACS2 with a *p*-value threshold of $1\times 10^{-6}$ . Additionally, SICERpy/0.1.1 (https://github.com/dariober/SICERpy, a wrapper for SICER from Zang et al., 2009) was used to call peaks for H3K27me3 using a gap size of 4 and a window size of 200bp. Paired-end (PE) reads were used for MACS2 peak calling, while only the first reads (R1) of the libraries were used for peak calling in SICERpy (Zang et al., 2009) as this software has yet to be optimized for PE libraries. The effective genome size for MACS2, or genome fraction for SICERpy, was determined by merging all input samples to identify the percentage of the genome covered by the merged bam file. The bioinformatic parameters used in peak calling for each mark are defined in Supplementary Table 3. DeepTools/3.5 (Ramírez et al., 2014) was employed to create combined signal tracks for each sample. Bam files were first scaled using signal extraction scaling (SES; Diaz et al., 2012) and input control signal was subtracted from each treatment sample. The signals from each biological replicate were then averaged for a given sample resulting in the final combined signal tracks.

### 2.5 Generating replicate-validated peak sets

Peak sets from each sample with an FE over input of greater than 2.0 for narrow marks, H3K27ac, H3K4me1, and H3K4me3, and 1.5 for broad marks, H3K27me3, were generated in Python/3.8. These FE-filtered peaks from one replicate were intersected with all called peaks from the other replicate using BEDTools/2.27.1 (Quinlan and Hall, 2010). The replicate-validated peaks from both replicates were merged to generate a combined peak set where all peaks achieved an FDR of less than 0.01 or 0.05, respective of histone mark, in both replicates and an FE of over 2.0 (or 1.5 for broad marks) in at least one replicate. These combined peak sets were used for tissue comparison in downstream analyses. The quality of the combined peak dataset was assessed by determining the Fraction of Reads in Peaks (FRiP) for each replicate in the corresponding peak file. FRiP scores were calculated using BEDTools/2.27.1 intersect by comparing the number of reads overlapping peaks to the total number of reads used for peak calling. BEDTools/2.27.1 was also employed to identify peaks unique to each tissue for a given histone mark. Microsoft Excel (Microsoft Corporation, 2018) was used to calculate Pearson’s correlations (*r*) between usable reads and total peak number, where usable reads is defined as the minimum number of filtered reads available for peak calling across biological replicates. Pearson’s correlations between total peak number and tissue-unique peak number were also assessed.

### 2.6 Feature annotation of combined peaks

Histone modification peaks were assigned to genomic features using the R package ChIPseeker/3.2 (Yu et al., 2015; Wang et al., 2022). A txdb annotation file was created from Ensembl’s EquCab3.0.113 using the R package txdbmaker/1.2.1 (Pagès et al., 2024). The annotatePeak function was employed with promoter region defined as ± 1000bp from the transcription start site (TSS).

## 3 Results

### 3.1 Sequencing depth and read filtration of paired-end libraries

On average, each stallion sample had 52 million (M) raw read pairs for H3K27ac and H3K4me1, 55 M for H3K4me3, and 134 M raw read pairs for H3K27me3. Filtering removed PCR duplicates, unmapped, and low-quality reads to create a set of read pairs used for peak calling, termed usable reads. The average number of usable reads was 28 M for H3K27ac, 32 M for H3K4me1, 30 M for H3K4me3, and 68 M for H3K27me3. Each tissue sample had an input sample with an average of 34 M reads used to remove background noise during peak calling for all marks. Despite generating over 215 M raw read pairs between the two H3K27ac_Adipose_AH3 libraries, less than 12 M usable reads were available for peak calling. Additionally, seven other sample/tissue combinations fell short of the targeted usable read counts; however, all were retained for analysis in the study regardless of read counts (Supplementary Table 4).

### 3.2 Quantifying peaks across tissues

**FIGURE 1:**
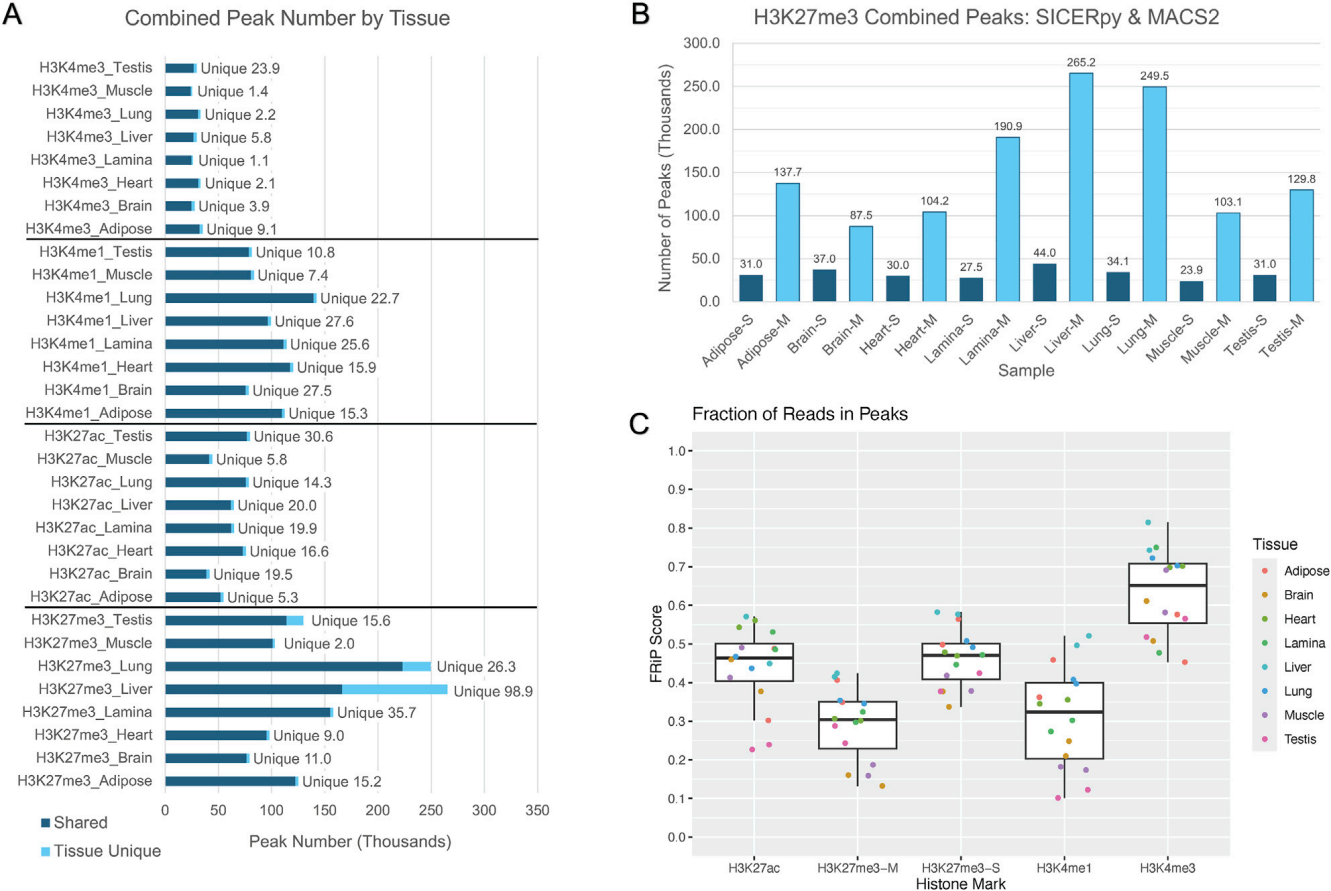
Peaks corresponding to histone modifications across the genome. **(A)** The bar plot depicts the number of peaks in thousands called by MACS2 corresponding to the signal from the denoted histone modifications: H3K4me3, H3K4me1, H3K27ac, and H3K27me3. The peaks identified to be unique to a given tissue for a given mark are discerned from shared peaks in light blue. **(B)** The peak calling software, MACS2 or SICERpy, results in a drastically different number of peaks being identified despite using the same input reads. Peaks called by SICERpy are in dark blue and labelled with an “-S,” while peaks called by MACS2 are light blue and labelled with “-M.” **(C)** The Fraction of Reads in Peaks (FRiP) score represents the number of read pairs from each replicate that fall within the combined peaks reported for a given tissue and mark. FRiP scores are separated by histone mark and peak calling software, if applicable. “-M” corresponds to MACS2 peaks and “-S” corresponds to SICERpy peaks. Each data point is colored to represent the tissue of origin for the given sample.

On average, each tissue had 76,778 H3K27ac peaks, 120,309 H3K4me1 peaks, and 33,969 H3K4me3 peaks (Figure 1A). Similar peak widths were observed across the narrow marks, with average peak widths of 1,360 bp, 1,219 bp, and 1,535 bp for H3K27ac, H3K4me1, and H3K4me3, respectively, yet peak width ranged considerably (Supplementary Figure 1). The number of peaks called for H3K27me3 varied based on the software used for peak calling. MACS2 identified an average of 158,480 H3K27me3 peaks while SICERpy called an average of 32,315 H3K27me3 peaks across tissues (Figure 1B). The average peak width of MACS2 H3K27me3 peaks was 1,650 bp while the average peak width of SICERpy H3K27me3 peaks was 18,466 bp (Supplementary Figure 1). The replicate-combined peaks captured read enrichment well with median FRiP scores of 0.46, 0.32, and 0.65 for H3K27ac, H3K4me1, and H3K4me3 peaks, respectively. The median FRiP scores for H3K27me3 peaks varied by peak caller with median FRiPs scores of 0.30 for MACS2 peaks and 0.47 for SICERpy peaks (Figure 1C). Although large difference in FRiP scores are observed between H3K27me3 peaks called by MACS2 and SICERpy, the difference in FriP scores and the difference in genome coverage are highly correlated (*r* = 0.94, data not shown).

H3K4me3 had the lowest genome coverage across tissues, averaging 2.2%. H3K27ac peaks covered an average of 4.1% of the genome across tissues, and H3K4me1 covered approximately 6.2% of the genome in each tissue. H3K27me3 peaks called by MACS2 covered an average of 10.4% of the genome, while H3K27me3 peaks called by SICERpy covered 24.3% of the genome in each tissue (Table 1). Despite the nearly two-fold greater genome coverage identified for H3K27me3 by SICERpy compared to MACS2, the peaks called by both software frequently overlapped. As a result of the smoothing effect of SICERpy, one large peak called by SICERpy often contained many smaller, consecutive peaks called by MACS2. In fact, 74%–94% of the peaks called by MACS2 overlapped those called by SICERpy (Supplementary Table 5).

**TABLE 1:** Percentage of the equine genome covered by histone marks in thoroughbred stallions.

Genome coverage (%) of histone marks					
Tissue	H3K27ac	H3K4me1	H3K4me3	H3K27me3-M^a^	H3K27me3-S^a^
Adipose	3.5	8.4	2.8	13.9	27.0
Brain	4.2	5.3	1.9	4.5	20.4
Heart	4.9	7.6	2.0	12.0	26.2
Lamina	4.1	5.7	1.6	11.3	24.0
Liver	4.9	8.8	2.1	17.4	31.9
Lung	4.4	7.9	2.0	11.2	24.4
Muscle	3.5	3.8	1.7	5.2	21.9
Testis	3.4	2.3	3.3	7.9	18.8
Average	4.1	6.2	2.2	10.4	24.3

^a^ H3K27me3-M refers to peaks called by MACS2 and H3K27me3-S corresponds to peaks called by SICERpy.

### 3.3 Genomic annotation of combined peaks

**FIGURE 2:**
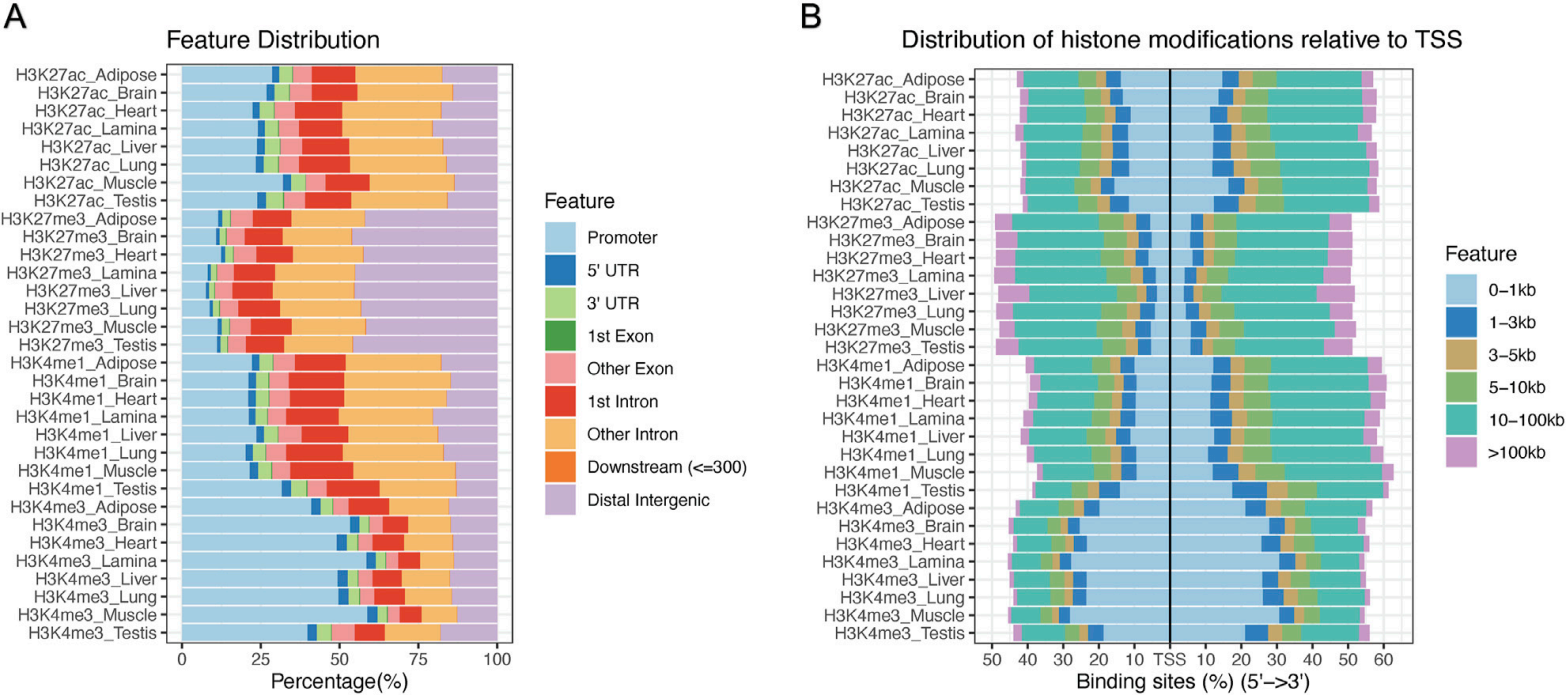
Distribution of histone modifications across the equine genome **(A)** The percentage of combined peaks assigned to gene features across samples. The promoter region is defined as ± 1000bp from the transcription start site (TSS), while downstream represents peaks within 300bp of the 3′ UTR of a given transcript. **(B)** The percentage of peaks falling within given distances of the TSS are presented. Known activating marks (H3K27ac and H3K4me3) fall closer to the TSS than the repressive mark, H3K27me3.

The combined MACS2 peaks for each tissue and histone mark combination were assigned to genomic features annotated in Ensembl’s 113 release of EquCab3.0. Due to the drastically increased peak widths of H3K27me3 peaks identified with SICERpy, averaging over 18kb, SICERpy H3K27me3 peaks were not annotated for genomic feature. H3K4me3 had the greatest number of peaks assigned to promoters ranging from approximately 35% in testis to 60% in muscle (Figure 2A). H3K27me3 had the fewest peaks assigned to promoters with over 40% of peaks identified as distal intergenic in all eight tissues. The distance between genomic features for a given transcript can vary dramatically based on heterogeneity in total transcript, exon, and intron lengths; therefore, distribution of peaks around the TSS is also reported (Figure 1B). Histone modifications commonly associated with gene activation (H3K4me3 and H3K27ac) are distributed more tightly around the TSS than the repressive mark, H3K27me3 (Figure 2B).

### 3.4 Tissue-unique peaks and correlations of usable reads and peak number

On average, 84% of peaks called for a histone mark were identified in more than one tissue, yet some tissues had a large percentage of unique peaks. The brain had the largest proportion of unique peaks for H3K4me1 and H3K27ac, with 27,532 peaks or 27% of H3K4me1 and 19,494 peaks or 33% of H3K27ac peaks identified as tissue-unique (Figure 1A; Table 2). Nearly 50% of H3K4me3 peaks in the testis were unique. The liver displayed the greatest proportion of unique peaks for H3K27me3 regardless of peak caller. Muscle consistently demonstrated low uniqueness across all four marks (Table 2). However, positive correlations existed between the number of reads used for peak calling and the number of peaks called. Correlation coefficients (r) between the minimum usable reads and total peaks called ranged from 0 to 0.86 across marks, with the smallest correlation observed in the H3K4me1 and the greatest correlation observed in H3K27me3 (Table 3). The number of unique peaks in a tissue was also highly correlated (0.52–0.92) with the total peaks called for that tissue (Table 2).

**TABLE 2:** Tissue-unique peaks and Pearson’s correlations of total and tissue-unique peak numbers.

Percentage (%) of peaks unique to tissue				
Tissue	H3K27ac	H3K4me1	H3K4me3	H3K27me3
Adipose	9.3	12.2	21.9	11.1
Brain	33.3	26.7	13.6	12.5
Heart	18.5	11.9	6.3	8.6
Lamina	24.3	18.7	4.3	18.7
Liver	24.5	22.2	17.7	37.3
Lung	15.9	14.0	6.7	10.5
Muscle	12.3	8.4	5.4	1.9
Testis	28.4	12.1	47.0	12.1
Pearson’s Correlation (*r*) Total:Unique Peaks	0.778	0.519	0.923	0.874

**TABLE 3:** Pearson’s correlations of minimum usable reads and replicate-validated combined peak number.

Mark	Sample	Replicate^a^	Minimum usable reads	Combined peaks	Pearson’s correlation
H3K27ac	Adipose	AH3	11,841,844	57,307	0.733
	Brain	AH3	28,767,074	58,463	
	Heart	AH4	28,986,609	89,654	
	Lamina	AH3	23,823,796	81,895	
	Liver	AH4	25,056,779	81,759	
	Lung	AH4	26,005,392	90,094	
	Muscle	AH3	16,216,664	47,415	
	Testis	AH4	31,632,424	107,633	
H3K4me1	Adipose	AH4	22,976,612	124,995	0.004
	Brain	AH4	33,748,599	103,254	
	Heart	AH3	31,026,046	133,481	
	Lamina	AH3	23,743,730	136,805	
	Liver	AH4	28,448,813	124,198	
	Lung	AH4	32,876,189	162,224	
	Muscle	AH3	29,981,650	87,973	
	Testis	AH4	27,586,751	89,543	
H3K4me3	Adipose	AH3	28,668,914	41,685	0.398
	Brain	AH3	30,245,100	28,804	
	Heart	AH4	32,088,317	33,200	
	Lamina	AH4	17,500,964	26,039	
	Liver	AH4	30,318,223	32,603	
	Lung	AH3	32,396,228	33,194	
	Muscle	AH3	19,685,919	25,333	
	Testis	AH3	27,740,548	50,893	
H3K27me3	Adipose	AH3	61,352,911	137,696	0.862
	Brain	AH3	51,495,389	87,453	
	Heart	AH3	46,128,360	104,207	
	Lamina	AH3	58,482,470	190,940	
	Liver	AH3	68,471,421	265,202	
	Lung	AH3	66,585,343	249,506	
	Muscle	AH3	43,455,906	103,055	
	Testis	AH3	44,709,318	129,784	

^a^ Replicate denotes which biological replicate had the fewest usable reads for the given sample.

### 3.5 Validation of tissue-specific epigenetic regulation

**FIGURE 3:**
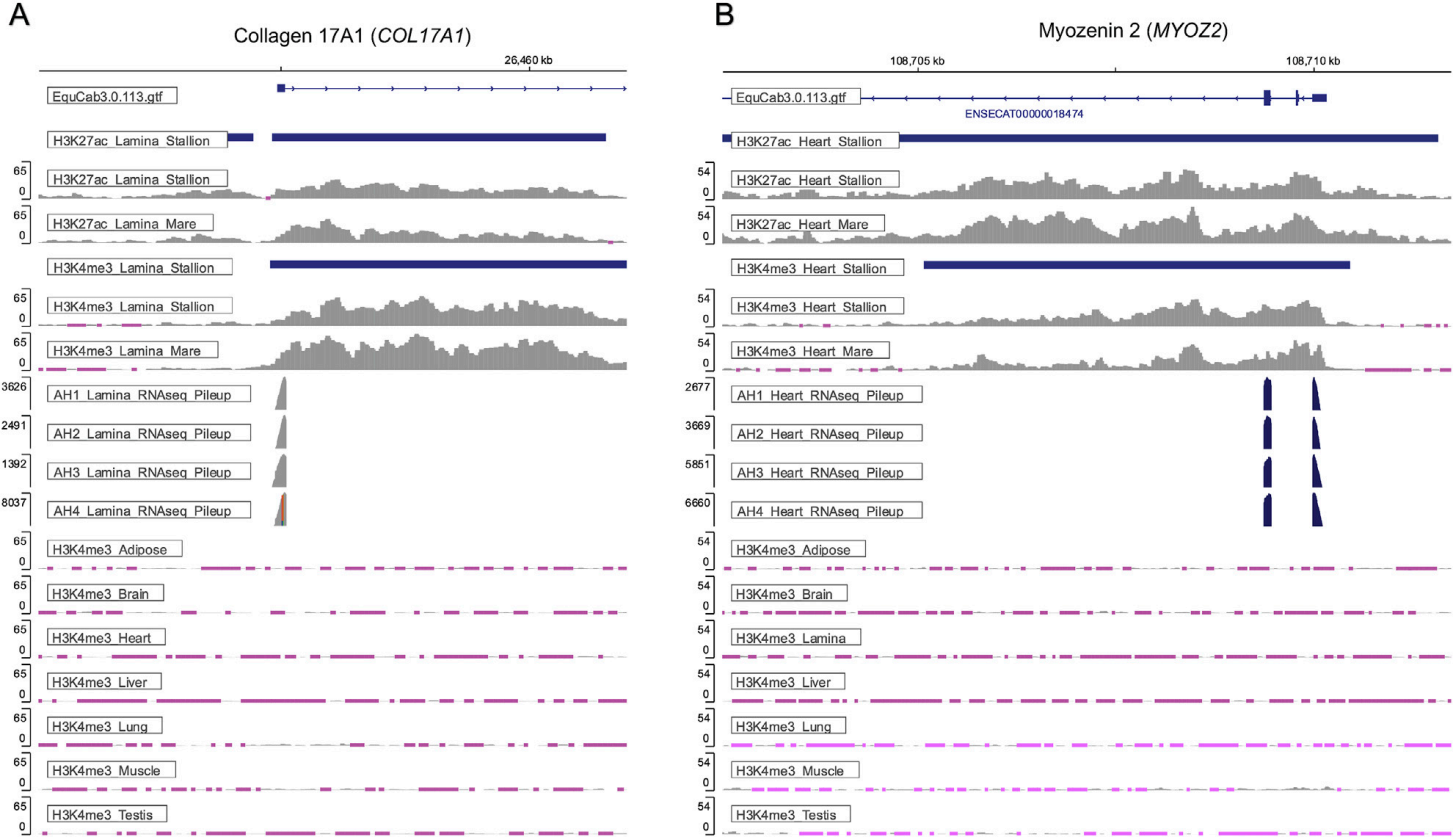
Tissue-specific active promoters are present across tissues and supported by gene expression data. **(A)** H3K4me3 and H3K27ac peaks within the promoter of the Collagen 17A1 (COL17A1) gene were uniquely identified in lamina from stallions. Enrichment of H3K4me3 under these peaks is only observed in lamina from the stallion tissues. Similar enrichment of H3K27ac and H3K4me3 are observed in lamina from mares (Kingsley et al., 2020), and the expression of COL17A1 can be confirmed by the RNA-seq data corresponding to lamina from the stallions in this study, as well as the lamina from the mares in Kingsley et al. (2020). **(B)** Enrichment of H3K4me3 and H3K27ac at the promoter of Myozenin 2 (MYOZ2) was uniquely observed in the heart tissue of stallions. Enrichment of these histone modifications is supported by the mare data from Kingsley et al. (2020), and gene expression of MYOZ2 is observed in the corresponding RNA-seq data from these tissues. All signal tracks depicted are available at https://equinegenomics.uky.edu. *Note: Pink regions of signal denote loci where the input signal is greater than the sample signal.

To assess how well tissue-unique peaks correspond to known tissue function, genes with unique active promoters, defined as having both H3K4me3 and H3K27ac peaks in their promoters, were identified. Examples for tissue-specific active promoters are provided in lamina at the Collagen 17A1 (*COL17A1*) gene (Figure 3A) and in heart at the Myozenin 2 (*MYOZ2*) gene (Figure 3B). The signal tracks present in Figure 3 demonstrate how the stallion ChIP-seq data can be integrated with mare ChIP-seq data (Kingsley et al., 2020) and the equine FAANG RNA-seq data to better assess tissue-specific epigenetic regulation.

## 4 Discussion

On average, each tissue in the stallions had over 250,000 peaks called across the four histone marks. The most common cis-regulatory associated histone modifications were H3K27me3, which play a role in repressing gene expression. This broad peak covered the greatest percentage of the genome, with some tissues having evidence of this repressive mark covering as much as 32% of the genome. This result differs from what was reported for mares with the greatest proportion of the genome covered (4.9%) in adipose as determined using SICERpy (Kingsley et al., 2020). On average, four times as many usable reads were available for H3K27me3 peak calling in stallion tissues compared to the mare tissues examined by Kingsley et al. (2020). A high correlation (*r* = 0.86) between usable reads and H3K27me3 peak number exists in the stallion tissues, suggesting the difference in genome coverage observed between sexes may be an artifact of sequencing depth. The H3K27me3 genome coverage of assessed stallion tissues is similar to other published data demonstrating that H3K27me3, and corresponding facultative heterochromatin, can stretch across 20%–30% of the genome under various circumstances (Hosogane et al., 2016; reviewed by Peng and Karpen, 2008). The enzymes that are involved in trimethylation of H3K27 often follow a positive feedback loop in which the presence of H3K27me3 increases trimethylation of nearby histones, which may explain the expansive genome coverage of H3K27me3 peaks in this study and others (Schmitges et al., 2011; Oksuz et al., 2018). Despite known biological variability of H3K27me3 across tissues, both the number and width of the H3K27me3 peaks identified in this study varied considerably based on the peak calling software. MACS2 identified nearly five times as many H3K27me3 peaks as SICERpy, yet the average width of the peaks called by SICERpy was over ten times larger than those called by MACS2. FRiP scores for H3K27me3 were high for both MACS2 and SICERpy peaks ranging from 0.10 to 0.58. These FRiP scores fall well above the ENCODE FRiP score threshold of 1% (Landt et al., 2012). Although SICERpy consistently produce greater FRiP scores than MACS2, the difference can almost exclusively be attributed to the difference in genome coverage between the peak calling software.

Steinhauser et al. (2016) also assessed peak calling software for broad peaks, including H3K27me3, in which SICER called considerably wider peaks than peak calling software based on MACS2. A gold standard method for peak calling has yet to be established, thus simulated ChIP-seq datasets are required to examine the sensitivity and specificity of peak calling software. On simulated datasets, SICER outperformed 10 different peak-calling tools for both identifying true peaks and limiting false positives when examining a broad-peaked histone modification (Steinhauser et al., 2016). SICER was designed to better capture broad and diffuse peaks, such as those of H3K27me3; therefore, peaks called by SICERpy may better represent the proportion of the genome repressed due to H3K27me3 (Xu et al., 2014). MACS2 is most often used to identify narrow peaks suggesting that H3K27me3 peaks called by MACS2 may represent regions of the genome with the strongest H3K27me3 signals.

The number of peaks called for each mark varied by tissue, yet muscle samples consistently had fewer peaks than other tissues across all histone marks possibly due to the lesser amount of chromatin retrieved for library preparation. However, the same amount of chromatin was used in the ChIP-seq analysis of mare tissue (Kingsley et al., 2020) with no reduction in peak number observed across skeletal muscle samples in that report. The muscle sample from one of the stallions (AH3) failed to produce the targeted number of usable reads for H3K27ac, H3K27me3, H3K4me3, and the input sample, yet in all cases, the number of usable reads was within 20% of the target. Even so, moderate positive correlations between the number of reads used for peak calling and the number of peaks called suggest that additional ChIP and/or sequencing may improve the identification of regulatory elements in muscle.

The strong positive correlation between the number of usable reads and peaks called for H3K27ac and H3K27me3 suggests that the ideal sequencing depth had not been reached in many of the samples. Although data from most tissues produced enough usable reads to achieve the thresholds established by ENCODE (https://www.encodeproject.org/chip-seq/histone/); 20 M for narrow marks and 45 M for broad marks), our data suggest that these thresholds may not be sufficient in all tissue types. This is well demonstrated by the broad mark, H3K27me3, when considering the shared tissues between mares and stallions. When increasing the average number of usable reads from 27 M in mares to 72 M in stallions, the average genome coverage of H3K27me3 peaks called by SICERpy increased from 3.8% to 25.1% (Kingsley et al., 2020). The need for additional sequencing beyond the guidelines set by ENCODE has also been echoed in other studies (Chen et al., 2012). Further work is necessary to determine at which point additional reads no longer enhance the ability to call peaks, which may differ across tissue types or due to tissue quality. It is important, however, that even if all regulatory elements in the assayed tissues were not captured, those that were served to annotate hundreds of thousands of cis-regulatory associated histone modifications, lending valuable information into the genome function of those tissues.

In addition to a moderate correlation between usable reads and peaks called, a strong positive correlation was identified between the number of peaks called in a tissue and those identified as unique to that tissue. This correlation makes it difficult to determine if these uniquely identified peaks represent biological differences in the regulatory elements of tissues or if they are an artifact of the total number of histone modifications captured across tissues. Yet, in the case of the H3K27ac and H3K4me1, the highest percentage of uniqueness is observed in the brain despite having fewer peaks than five of the other tissues. Similarly, the highest percentage of unique peaks occurred for H3K27ac in brain of mares as previously reported by Kingsley et al. (2020). Inevitably, the tissue-unique peaks identified will vary as additional tissues are examined; however, many identified tissue-unique histone modifications marked genes with known, tissue-specific functions. Examples in which unique activating marks are found include collagen 17A1 (*COL17A1*) in the lamina and Myozenin 2 (*MYOZ2*) in the heart. *COL17A1* is enriched in skin in humans and functions in maintaining the epidermal-dermal junction (NIH GeneID: 1,308). *MYOZ2* was shown to have tissue-specificity in human cardiac myocytes (NIH GeneID: 51,778). The H3K27ac and H3K4me3 signals in these genes in lamina and heart, respectively, are well supported by the ChIP-seq data in mares (Kingsley et al., 2020), and further supported by high expression of *COL17A1*in lamina and *MYOZ2* in the left ventricle of the heart in corresponding RNA-seq data. Although further work to validate tissue-unique peaks will need to involve additional tissue analysis and corresponding transcriptomics data, preliminary analysis confirms that many tissue-specific histone modifications identified in this manuscript are supported by previously published data.

The annotation of regulatory elements has proven beneficial in characterizing the function of the genome and associating genomic variation with disease in humans (The ENCODE Project Consortium, 2012; Adkemir et al., 2020; van der Lee et al., 2020; Claringbould and Zaugg, 2021; Zhang et al., 2022). In this study, hundreds of thousands of cis-regulatory associated histone modifications were identified across tissues in the Thoroughbred stallion, providing foundational information into the function of the equine genome. The data from the previously published ChIP-seq analyses in the mares has already aided in the identification of variants associated with distichiasis and the characterization of centromere sliding in horses (Kingsley et al., 2020; Hisey et al., 2020; Cappelletti et al., 2023). The ChIP-seq analyses in the stallion provide additional support for the annotation of regulatory elements present in the tissues of adult horses and may be valuable in determining differences in epigenomic regulation across sex in horses. Although peaks unique to tissues in this study cannot entirely be attributed to true biological differences, they provide a basis for hypothesis generation and testing. These analyses demonstrate some of the shortcomings in the current methodology and standards used for identifying cis-regulatory elements. As previously suggested, chromatin extraction, library preparation, sequencing methods, and peak calling software have large impacts on the interpretation of ChIP-seq experimental data (Steinhauser et al., 2016; Zhang et al., 2016; Nakato and Shirahige, 2017; Xiang et al., 2020). These artifacts of data processing can impair the ability to accurately identify biological differences across datasets. While much progress has been made in our understanding of genome function with the annotation of cis-regulatory associated histone modifications, technological advancements will be necessary for enhanced comparative studies of genome regulation across sexes and species.

## Data Availability

The datasets presented in this study can be found in online repositories. The names of the repository/repositories and accession number(s) can be found below: https://www.ncbi.nlm.nih.gov/bioproject/PRJEB57637/; https://equinegenomics.uky.edu. Processed data, including individual narrow Peak files and annotated replicate-verified BED files, are available on Open Science Framework at osf.io/ghvp9 (Barber, 2025).
