# Supplementary material for: Annotation of cis-regulatory-associated histone modifications in the genomes of two Thoroughbred stallions
Source: Front Genet. 2025 Feb 27;16:1534461. doi: 10.3389/fgene.2025.1534461 (PMC11903428; doi:10.3389/fgene.2025.1534461)
Supplement: Supplementary file 1 [file DataSheet1.pdf]

Supplementary Table 1. Parameters for ChIP Extraction and Shearing

	Adipose	Brain	Heart	Lamina	Liver	Lung	Muscle	Testis
Starting Tissue (mg)	208	109	103	95	55	53	106	105
Homogenization Time (min)	8	5	5	5	5	5	5	5
Fixation Time (min)	10	9	9	9	9	9	9	12
Shearing Volume (µL)	400	1500	1800	1800	1500	1500	1500	1300
Shearing Cycles	5 x 8[1]	10	10	10	8	10	10	8
Chromatin per IP (ng)	200	700	800	900	1500	1500	280	1500

[1]Chromatin from adipose was sheared five times for 8 cycles

Supplementary Table 2. Chromatin and Antibody Amounts Used for ChIP of Stallion Tissues

**Tissue**	**Chromatin IP (ng)**	**Antibody Amount (µg)**			
		**H3K27ac**	**H3K4me1**	**H3K4me3**	**H3K27me3**
**Adipose**	200	1	0.5	0.5	1
**Brain**	700	0.5	0.5	0.5	1
**Heart**	800	0.5	0.5	0.5	1
**Lamina**	900	1	0.5	0.5	1
**Liver**	1500	2	1	1	2
**Lung**	1500	2	1	1	1
**Muscle**	280	1	0.5	0.5	0.5
**Testis**	1500	2	1	1	2

Supplementary Table 3. Software Parameters for ChIP-seq Peak Calling in MACS2 and SICER

Software	Parameter	H3K27ac	H3K4me1	H3K4me3	H3K27me3
MACS2	Size	Narrow	Narrow/ Intermediate	Narrow	Broad
	Size Flag	n/a			--broad
	FDR[1]	0.01	0.05	0.01	0.05
	Broad Cutoff	n/a			0.1
	Genome size	2,409,159,894 bp			
SICERpy[2]	Gap Size	n/a			4
	Window Size	n/a			200
	Genome Fraction	n/a			0.973

[1]The false discovery rate (FDR) cutoff dictates both peak number and peak width in MACS2, so histone marks with broader peaks have looser FDR cutoffs (https://github.com/hbctraining/Intro-to-ChIPseq). H3K4me1 was at one point considered to have broad peaks but has since been determined to have an intermediate peak width (Kingsley et al., 2020).
[2]SICER was only used to call peaks for the broad mark, H3K27me3.

Supplementary Table 4. The Number of Raw Read Pairs Generated and Usable Read Pairs Remaining After Filtering for Stallion Samples

Tissue_Replicate	H3K27ac		H3K4me1		H3K4me3		H3K27me3		Input	
	Raw Read Pairs	Usable Read Pairs	Raw Read Pairs	Usable Read Pairs	Raw Read Pairs	Usable Read Pairs	Raw Read Pairs	Usable Read Pairs	Raw Read Pairs	Usable Read Pairs
Adipose_AH3*	68,730,027	**9,161,533**	87,399,621	44,863,661	53,728,350	28,668,914	163,649,254	61,352,911	57,287,919	33,441,186
Adipose_AH4	64,121,448	**19,628,311**	73,755,325	22,976,612	71,588,828	30,633,260	172,819,418	77,353,465	56,710,987	31,577,565
Brain_AH3	44,153,723	28,767,074	51,902,716	39,353,133	48,165,691	30,245,100	89,113,219	51,495,389	43,318,326	30,758,728
Brain_AH4	53,550,924	37,164,559	45,941,617	33,748,599	44,199,605	31,024,168	123,828,178	70,346,101	45,370,082	29,345,385
Heart_AH3	51,902,076	32,672,638	43,860,168	31,026,046	51,509,237	33,775,014	115,749,332	46,128,360	43,193,521	31,251,565
Heart_AH4	45,090,183	28,986,609	64,302,815	36,586,039	44,069,238	32,088,317	141,578,920	65,737,749	59,163,545	41,930,841
Lamina_AH3	40,535,808	23,823,796	51,986,630	23,743,730	48,277,375	24,570,676	91,980,974	58,482,470	44,456,575	32,290,294
Lamina_AH4	51,953,800	24,590,690	49,361,910	29,682,378	41,990,329	**17,500,964**	175,850,495	107,502,509	57,425,242	38,117,196
Liver_AH3	55,697,969	35,491,407	45,896,092	30,860,281	69,512,707	39,275,132	126,294,140	68,471,421	66,316,277	44,083,269
Liver_AH4	46,094,286	25,056,779	40,668,291	28,448,813	58,847,906	30,318,223	192,622,865	102,596,377	51,996,032	36,922,319
Lung_AH3	51,223,693	36,118,820	54,431,763	38,105,392	45,017,929	32,396,228	120,694,401	66,585,343	50,253,855	36,332,494
Lung_AH4	38,186,321	26,005,392	46,855,312	32,876,189	48,153,388	33,423,330	174,764,785	85,474,365	44,634,249	31,783,927
Muscle_AH3	59,556,353	**16,216,664**	50,633,437	29,981,650	71,090,388	**19,685,919**	80,658,715	**43,455,906**	43,227,165	**18,252,957**
Muscle_AH4	59,556,353	34,276,737	50,633,437	34,362,506	71,090,388	31,289,430	211,037,887	99,616,774	43,227,165	30,297,899
Testis_AH3	59,238,170	42,301,940	42,281,966	29,481,648	56,479,066	27,740,548	80,574,901	**44,709,318**	49,793,842	36,030,620
Testis_AH4	44,952,694	31,632,424	38,900,952	27,586,751	51,773,961	34,079,183	88,305,332	45,983,224	54,636,399	36,616,903
Adipose_AH3_ Reseq*	146,616,053	**2,680,311**	-	-	-	-	-	-	-	-
Adipose_AH3_ Merged*	215,346,080	**11,841,844**	-	-	-	-	-	-	-	-

*The "Usable Read Pairs" from both the original and resequenced (Reseq) H3K27ac_Adipose_AH3 samples were merged prior to peak calling.

** The bolded cells represent samples that did not reach the usable read pair target of 45 M for H3K27me3 and 20 M for the remaining marks and input samples.

Supplementary Table 5. Overlap of MACS2 H3K27me3 Peaks with SICERpy H3K27me3 Peaks

	Total MACS2 Peaks	Overlapping MACS2 Peaks	Percentage of Overlapping Peaks
**Adipose**	137,696	119,044	86%
**Brain**	87,453	74,844	86%
**Heart**	104,207	89,129	86%
**Lamina**	190,940	149,917	79%
**Liver**	265,202	212,970	80%
**Lung**	249,506	206,519	83%
**Muscle**	103,055	96,847	94%
**Testis**	129,784	95,889	74%

A

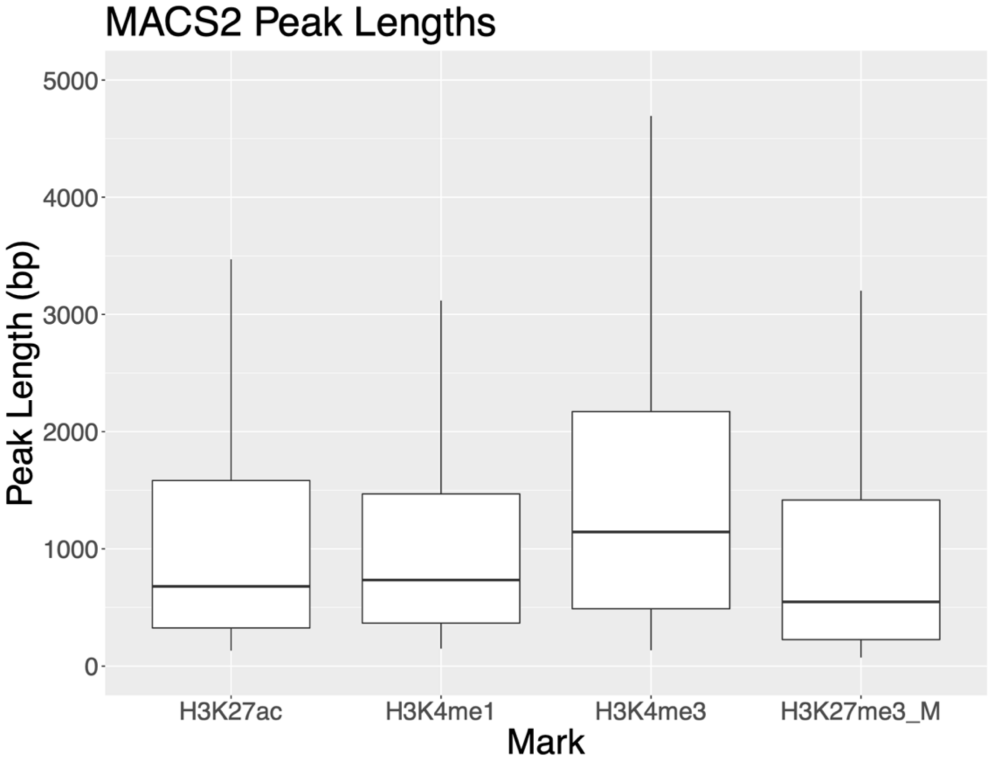

B

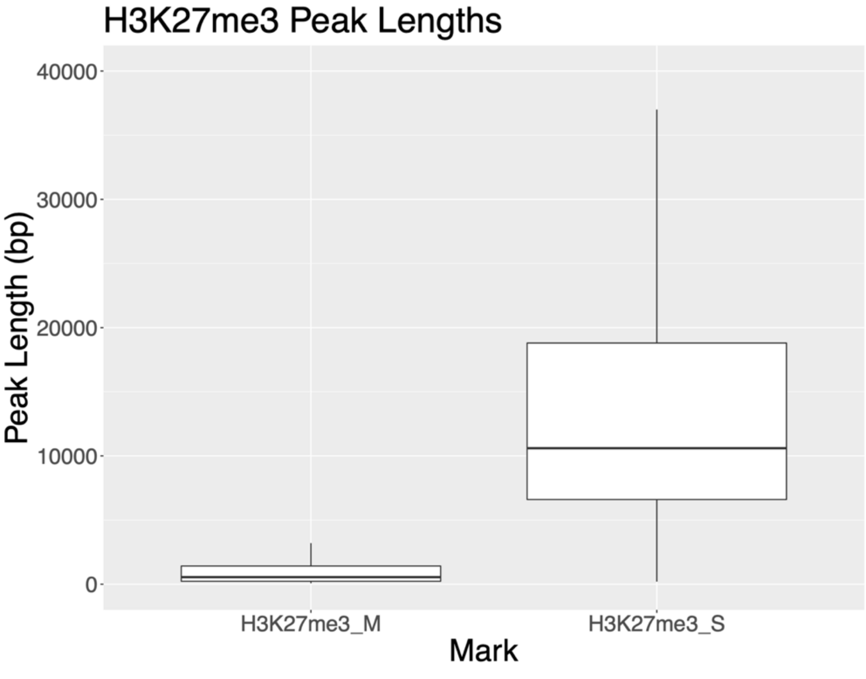

Supplementary Figure 1. Peak Length Distribution by Histone Mark. The distribution of peak length called by MACS2 was similar across all four histone marks (A), yet the distribution of peak lengths for H3K27me3 varied drastically between peaks called by MACS2 and SICERpy (B).
